# 

**Published:** 1915-12-11

**Authors:** 


### PASSING EVENTS AND TOPICS.

### Death of a Medical Sub-Editor.

We regret to learn that our esteemed contemporary the *Lancet* has sustained a loss by the death of Mr. Kenneth William Millican, B.A. (Cantab.), M.R.C.S., L.R.C.P., who held the position of sub-editor.

### The Lord Mayor and Hospital Funds.

The Lord Mayor of London has accepted the presidency of the Hospital Sunday and Hospital Saturday Funds during his year of office. He has also become a member of the Council of King Edward's Hospital Fund for London.

### Gift from Scottish Bowlers.

The Scottish Bowling Association has presented four more motor ambulances to the Red Cross Society. This brings the number contributed by this Association to ten; six motor ambulances having been previously presented to the Scottish branch of the Red Cross.

### Of Interest to Nurses.

It is notified by the War Office that nurses holding certificates for three years' general training who are desirous of being employed in military hospitals should apply in writing, without delay, to the Matron-in-Chief, Q.A.I.M.N.S., War Office, Whitehall, S.W., for conditions of service.

### Holly for War Hospitals.

The happy suggestion has been made by a Dorset correspondent of the *Daily Telegraph* that a central depôt should be established for receiving and distributing holly for war hospitals. The country folk would be delighted to send to such a depôt this ever-welcome and beautiful Yuletide tribute.

### Royal Surgical Aid Society.

The fifty-third annual meeting of the Royal Surgical Aid Society was held at the Mansion House on December 7. The Lord Mayor, who presided, said that during the past year 25,471 persons were benefited, and 39,290 appliances distributed. As a result of the meeting £318 was contributed.

### St. Bartholomew's Hospital and the Wounded.

The War Office recently applied to the governors of St. Bartholomew's Hospital for further accommodation, but as it was scarcely possible to give up any more of the civilian wards, the convalescent home at Swanley, with its seventy beds, was offered and accepted as a substitute. The first patients arrived on December 3.

### "Fee-Splitting" in America.

Three years ago the Missouri State Medical Association adopted a by-law against the practice known in the United States by the expressive name of "fee-splitting," but the first offence against this by-law has only just been reported. The offender, who was found guilty by the Board of Censors, has been expelled from the Association.

### The Shortage of Nitrous Oxide Gas.

At the annual meeting of the Incorporated Dental Hospital of Dublin it was stated that for some time in the summer it was found quite impossible to obtain a supply of nitrous oxide gas. The difficulty, however, appears to have been overcome, both in Ireland and elsewhere, for we have heard no serious complaints that the supplies of this gas are now inadequate.

### Claret for Soldiers.

The Under-Secretary for War is apparently unfamiliar with the belief that is common in wine-drinking countries that the acid in the wine tends to render those who drink it less liable to certain diseases. In the House of Commons on Monday he threw ridicule on the idea that if claret were served out to the British troops on the Gallipoli Pensinsula it might prove salutary as a preventive of disease. Nevertheless, it is said that it was on the advice of a commission of eminent French medical men that French wines are supplied to the French troops.

### Glasgow Lock Hospital.

The directors have now opened a dispensary at the Lock Hospital at 45 Rottenrow, for the treatment of outdoor patients suffering from the various forms of venereal disease. Structural alterations of the buildings have been completed, providing waiting-room accommodation and a fully-equipped treatment room. The Lock Hospital, or Hospital for Women as the patients prefer to call it, is the second oldest medical institution in Glasgow, having been founded by Seal of Cause from the magistrates and Town Council in 1805, ten years after the opening of the Royal Infirmary.

### Gifts and Bequests.

Mr. and Mrs. John Stansfield, of Osborne Grove, Bromley, have given £1,000 to the Victoria Hospital in that town to endow a bed in memory of their son, Second-Lieutenant Frank Stansfield, who was killed in the Dardanelles.

Mr. W. H. Bartholomew, of Headingley, Leeds, has made another gift to the Goole Bartholomew Hospital, of which he is the president, having donated to the committee of management £1,000 invested in War Stock to provide for the repair and upkeep of the extension of the buildings. This brings the amount of Mr. Bartholomew's gifts to the hospital to £13,000.

RATES OF SUBSCRIPTION: Twelve months, 6/6; Six months, 4/-; Three months, 2/-, post free to any address in the United Kingdom. Abroad, Twelve months, 8/8; Six months, 5/-; Three months, 2/6, post free.—Address The Subscription Dept., "THE HOSPITAL," The Hospital Building, 28/29 Southampton Street, Strand, London, W.C.

